# Retinal Vessel Oxygen Saturation during 100% Oxygen Breathing in Healthy Individuals

**DOI:** 10.1371/journal.pone.0128780

**Published:** 2015-06-04

**Authors:** Olof Birna Olafsdottir, Thorunn Scheving Eliasdottir, Jona Valgerdur Kristjansdottir, Sveinn Hakon Hardarson, Einar Stefánsson

**Affiliations:** 1 Department of Ophthalmology, University of Iceland, Reykjavik, Iceland; 2 Department of Ophthalmology, Landspitali—The National University Hospital of Iceland, Reykjavik, Iceland; Indiana University College of Medicine, UNITED STATES

## Abstract

**Purpose:**

To detect how systemic hyperoxia affects oxygen saturation in retinal arterioles and venules in healthy individuals.

**Methods:**

Retinal vessel oxygen saturation was measured in 30 healthy individuals with a spectrophotometric retinal oximeter (Oxymap T1). Oximetry was performed during breathing of room air, 100% oxygen (10 minutes, 6L/min) and then again room air (10 minutes recovery).

**Results:**

Mean oxygen saturation rises modestly in retinal arterioles during 100% oxygen breathing (94.5%±3.8 vs. 92.0%±3.7% at baseline, p<0.0001) and dramatically in retinal venules (76.2%±8.0% vs. 51.3%±5.6%, p<0.0001). The arteriovenous difference decreased during 100% oxygen breathing (18.3%±9.0% vs. 40.7%±5.7%, p<0.0001). The mean diameter of arterioles decreased during 100% oxygen breathing compared to baseline (9.7±1.4 pixels vs. 10.3±1.3 pixels, p<0.0001) and the same applies to the mean venular diameter (11.4±1.2 pixels vs. 13.3±1.5 pixels, p<0.0001).

**Conclusions:**

Breathing 100% oxygen increases oxygen saturation in retinal arterioles and more so in venules and constricts them compared to baseline levels. The dramatic increase in oxygen saturation in venules reflects oxygen flow from the choroid and the unusual vascular anatomy and oxygen physiology of the eye.

## Introduction

The vascular anatomy of the eye is unusual in that many ocular tissues are avascular or partially vascular and consequently, the oxygen physiology is extraordinary. The retina is partially vascularised and served by two blood circulations, the retinal circulation intrinsic to the tissue and the choroidal circulation adjacent to the outer retina. The choroidal circulation supplies the outer retina with oxygen whereas the inner retina is oxygenated by the retinal circulation.

The retinal circulation responds readily to changes in perfusion pressure as well as to oxygen tension by autoregulatory response [1]. This intrinsic capacity of the arteriolar vessel wall regulates arteriolar diameter and thereby controls retinal blood flow and oxygen delivery to the retinal tissue. It also results in an unusual response to increased oxygen breathing, compared to most other tissues.

Hyperoxia, such as with 100% oxygen breathing, does not happen in nature and only as a consequence of human intervention. In general, breathing 100% oxygen increases the oxygen content of blood by only about 10%. Fully oxygenated hemoglobin carries about 200 ml of oxygen in one liter of blood [2] and about 24 ml of oxygen/liter of blood per atmosphere can be carried dissolved in water [3]. The 10% increase in oxygen content going from 21% oxygen breathing to 100% oxygen breathing has a modest effect on oxygenation in most tissues. In most organs, including the central nervous system, a considerable amount of oxygen bound to haemoglobin is delivered into the tissue. This is different in the eye, particularly in the choroidal circulation. The blood flow in the choroidal circulation is so high under normal conditions that normally only about 3% of the oxygen content is extracted and delivered to the outer retina [4]. The choroid also shows limited blood flow response to 100% oxygen breathing [5], which is different from the retina where vasoconstriction and decreased blood flow has repeatedly been measured during pure oxygen breathing in humans [6–8]. During pure oxygen breathing, the oxygen tension (PO_2_) of the choroid remains high and the choroid delivers a large flux of oxygen to all parts of the retina. The entire amount of oxygen needed by the outer retina can be supplied from oxygen dissolved in choroidal serum during 100% oxygen breathing and a large oxygen flux reaches the inner retina. This has been demonstrated repeatedly in experimental animals [9–11] but technical limitations have made studies of this difficult in humans until now.

We have developed a spectrophotometric retinal oximeter which is based on a fundus camera and allows for safe non-invasive measurement of retinal vessel oxygen saturation as well as retinal vessel diameter in human subjects. This allows us to study the effect of 100% oxygen breathing on the oxygen metabolism of the human retina.

## Methods

### Ethics statement

The study was approved by the National Bioethics Committee of Iceland and The Icelandic Data Protection Authority and adhered to the tenets of the Declaration of Helsinki. All participants signed an informed consent.

### Subjects

Out of 33 healthy individuals that participated in the study, 30 individuals were included in the analysis (19 females, 11 males; mean age: 44±18 years). Inclusion criteria consisted of a healthy eye with no ocular disease. Exclusion criteria consisted of smoking, any eye disease and any systemic diseases that could affect the eye or oxygen levels such as diabetes, respiratory and cardiovascular disease. It was required that end tidal oxygen stability (plateau) was reached during 100% oxygen breathing. Three individuals were excluded from the healthy group, one individual was suspected of having glaucoma and the other two did not achieve end tidal oxygen stability during breathing of 100% oxygen. All participants had been examined by an ophthalmologist no more than seven months prior to the study.

A standard study protocol was followed. Each individual answered a questionnaire on medical history, medications and smoking followed by measurement of intraocular pressure (IOP) (iCare TAO1 Tonometer, Tiolat Oy, Helsinki, Finland). Before oximetry measurements. both eyes were dilated with 1% tropicamide (Mydriacyl; S.A. Alcon-Couvreur N.V., Puurs, Belgium).

### Retinal Oximetry

The retinal oximeter Oxymap T1 (Oxymap ehf., Reykjavik, Iceland) has been described in detail elsewhere [12]. It is composed of two digital cameras (Insight IN 1800, 1600 x 1200 square pixels, Diagnostic Instruments Inc., Mi, USA), a custom made adapter, an image splitter and two narrow band-pass filters. It is attached to a fundus camera (Topcon TRC-50DX, Topcon Corporation, Tokyo, Japan) and simultaneously yields two fundus images of the same area of the retina at two different wavelengths of light, 570 nm, which is insensitive to oxygen saturation and 600 nm, which is sensitive to oxygen saturation.

A pseudocolor fundus map is automatically generated with specialized software (Oxymap Analyzer software 2.2.1, version 3847, Oxymap ehf., Reykjavik, Iceland). The software selects measurement points on the fundus image and calculates optical density of retinal vessels at the two wavelengths. The ratio of the optical densities at the two wavelengths is sensitive to oxygen saturation and has an inverse and approximately linear relationship to oxygen saturation [13, 14].

**Fig 1:**
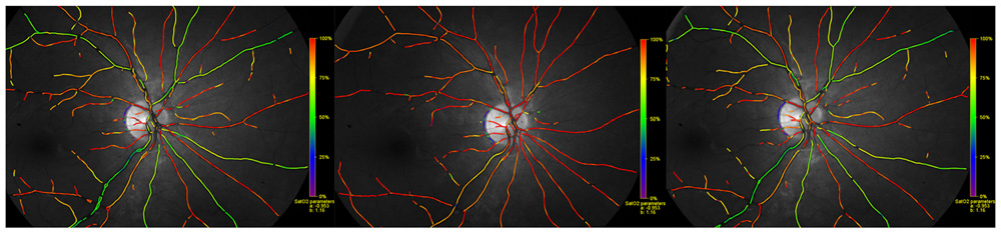
A pseudocolor fundus map of oxygen saturation during A. Baseline (room air breathing), B. 100% oxygen breathing for ten minutes and C. Recovery (room air breathing for 10 minutes after 100% oxygen breathing). Red color denotes oxygen saturation approximately 90–100% and green color denotes oxygen saturation approximately 50–60% (see scale on right side of each image).

Oximetry was performed three times for each eye; (1) prior to inhalation of 100% oxygen (baseline), (2) after 10 minutes of inhalation of 100% oxygen and (3) after 10 minutes of breathing room air (recovery). On each image of the fundus, the optic nerve head was located at the center (Fig 1). Oxygen saturation was calculated as a mean for all arterioles and venules for each image of the right eye. The measured vessels segments were six pixels or more in diameter, between 50–200 pixels in length and could be carefully paired between all three images (one pixel equals approximately nine micrometers). The measured vessels were all first degree vessels or second degree if the length of the first degree was <50 pixels. An area of 15 pixels was excluded around the optic disc. When the face mask was removed, subjects did not inhale room air until oximetry images had been acquired.

In the case of two healthy subjects (two males of 24 and 33 years of age), images were acquired every five seconds during a period of 120 seconds, which started when breathing of 100% oxygen stopped and breathing of room air began (recovery from hyperoxia).

### Gas breathing system

A soft cushion inflatable face mask (Flexicare, Flexicare Medical Ltd., Mountain Ash., UK), connected to circle system with carbon dioxide absorber of an anesthesia machine (Dameca: Siesta 10770, Roedovre, Denmark), was placed over the mouth and nose of the subject´s face. Head strap was attached to the retaining hooks surrounding the facial mask orifice to create an airtight seal which was further supported by the participant´s hand. The flow of oxygen was set to 6 L/min and 100% oxygen was inhaled for 10 minutes.

### Continuous measurements of physiological variables

Brachial artery blood pressure (Omron M6 Comfort (HEM-7221-E) Omron Healthcare Europe, Hoofddorp, The Netherlands) was obtained three times; before inhalation of 100% oxygen, when 100% oxygen had been inhaled for nine minutes and again when subjects had recovered for 10 minutes by breathing room air. The following parameters were continuously monitored using a gas analyzer (Datex-Ohmeda D-LCC15.03, Planar Systems Inc., Beaverton Oregon, USA): respiratory rate (RR): end-tidal carbon dioxide (ETCO_2_), fraction of inhaled carbon dioxide (FICO_2_), concentration of inhaled oxygen (FIO_2_) and end-tidal oxygen (ETO_2_). Systemic oxygen saturation and heart rate were monitored with a finger pulse oximeter (SpO_2_) (Datex- Ohmeda OxyTip+ Healthcare, Finland) (see Table 1).

**Table 1:** Physiological variables that were measured (mean± SD) during room air breathing (Baseline), 100% oxygen breathing and 10 minutes of room air breathing subsequent to 100% oxygen breathing (Recovery).

Physiological variables	Baseline	100% O_2_ Breathing	Recovery
EtCO_2_ (mmHg)	-	37.5±3.4	-
HR (beats/min)	72±11	70±11	-
IOP (mm Hg)	15±4	-	-
BP_systolic_ (mmHg)	132±20	127±22	128±19
BP_diastolic_ (mmHg)	84±12	86±14	84±14
MAP (mm Hg)	100±14	100±15	99±15
OPP (mm Hg)	52±10	-	-
SpO_2_ (pulse oximetry, %)	97.5±0.7	99.1±0.3	-

EtCO_2_, end tidal carbon dioxide; HR, heart rate; IOP, intraocular pressure; BP, blood pressure; MAP, mean arterial pressure; OPP, ocular perfusion pressure; SpO_2_, finger oxygen saturation.

Mean arterial pressure (MAP) was calculated as MAP = 2/3DP+1/3SP. Ocular perfusion pressure (OPP) was calculated as 2/3MAP-IOP.

Arteriovenous (AV) difference was calculated by oxygen saturation in venules subtracted from oxygen saturation in arterioles.

### Statistics

Statistical analysis was performed with Prism, version 5.01 (GraphPad Software Inc., LaJolla, CA, USA). Paired Student’s t-test was used where p<0.05 was considered statistically significant.

## Results

**Fig 2:**
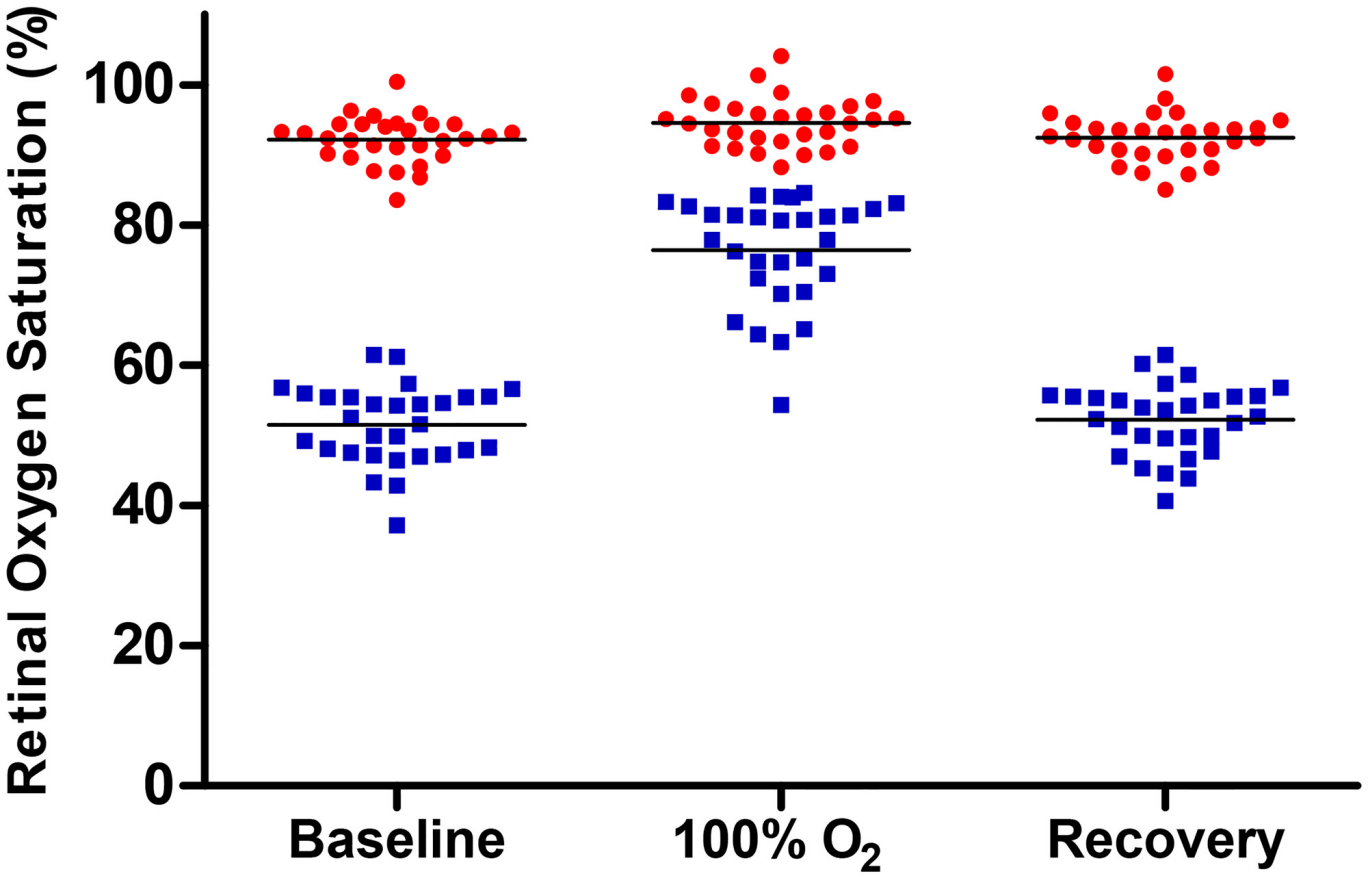
Retinal vessel oxygen saturation in arterioles and venules; before 100% oxygen breathing (baseline), during 100% oxygen breathing and 10 minutes after 100% oxygen breathing (recovery) in healthy individuals (n = 30). The lines show mean values for each group. Each point denotes value from one individual.

Retinal vessel oxygen saturation was elevated during 100% oxygen breathing compared to baseline for arterioles (94.5%±3.8% vs. 92.0%±3.7%, p<0.0001) and venules (76.2%±8.0% vs. 51.3%±5.6%, p<0.0001)). AV difference was statistically significantly lower during 100% oxygen breathing compared to baseline (18.3%±9.0% vs. 40.7%±5.7%, p<0.0001). All oxygen saturation values are presented in Table 2 and Fig 2.

**Table 2:** Retinal vessel oxygen saturation during baseline, 100% oxygen breathing and recovery (10 minutes of room air breathing after 100% oxygen breathing).

	Baseline	100% Oxygen Breathing	Recovery	Baseline vs. 100% O_2_ Breathing
**Oxygen saturation (%, Mean ± SD)**				
Arterioles	92.0 ± 3.7	94.5 ± 3.8	92.3 ± 3.5	p<0.0001
Venules	51.3 ± 5.6	76.2 ± 8.0	51.5 ± 5.5	p<0.0001
AV-difference	40.7 ± 5.7	18.3 ± 9.0	40.8 ± 5.9	p<0.0001
**Diameter (Pixels, Mean ± SD)**				
Arterioles	10.3±1.3	9.7±1.4	10.2±1.3	p<0.0001
Venules	13.3±1.5	11.4±1.2	13.1±1.4	p<0.0001

The p-values show statistical difference between baseline and 100% oxygen breathing (Student´s t-test).

The arteriolar diameter decreased during 100% oxygen breathing compared to normoxia at baseline (9.7±1.4 pixels vs. 10.3±1.3 pixels, p<0.0001). The diameter of the venules also decreased (11.4±1.2 pixels vs. 13.3±1.5 pixels, p<0.0001).

There was no difference in oxygen saturation between baseline and recovery and this applies to arterioles, venules and AV difference (p = 0.2–0.8, Table 2). There was also no difference in arteriolar diameter between baseline and recovery (p = 0.3). The venular diameter is slightly smaller after recovery compared to baseline (13.1±1.4 vs. 13.3±1.5, p = 0.007).

**Fig 3:**
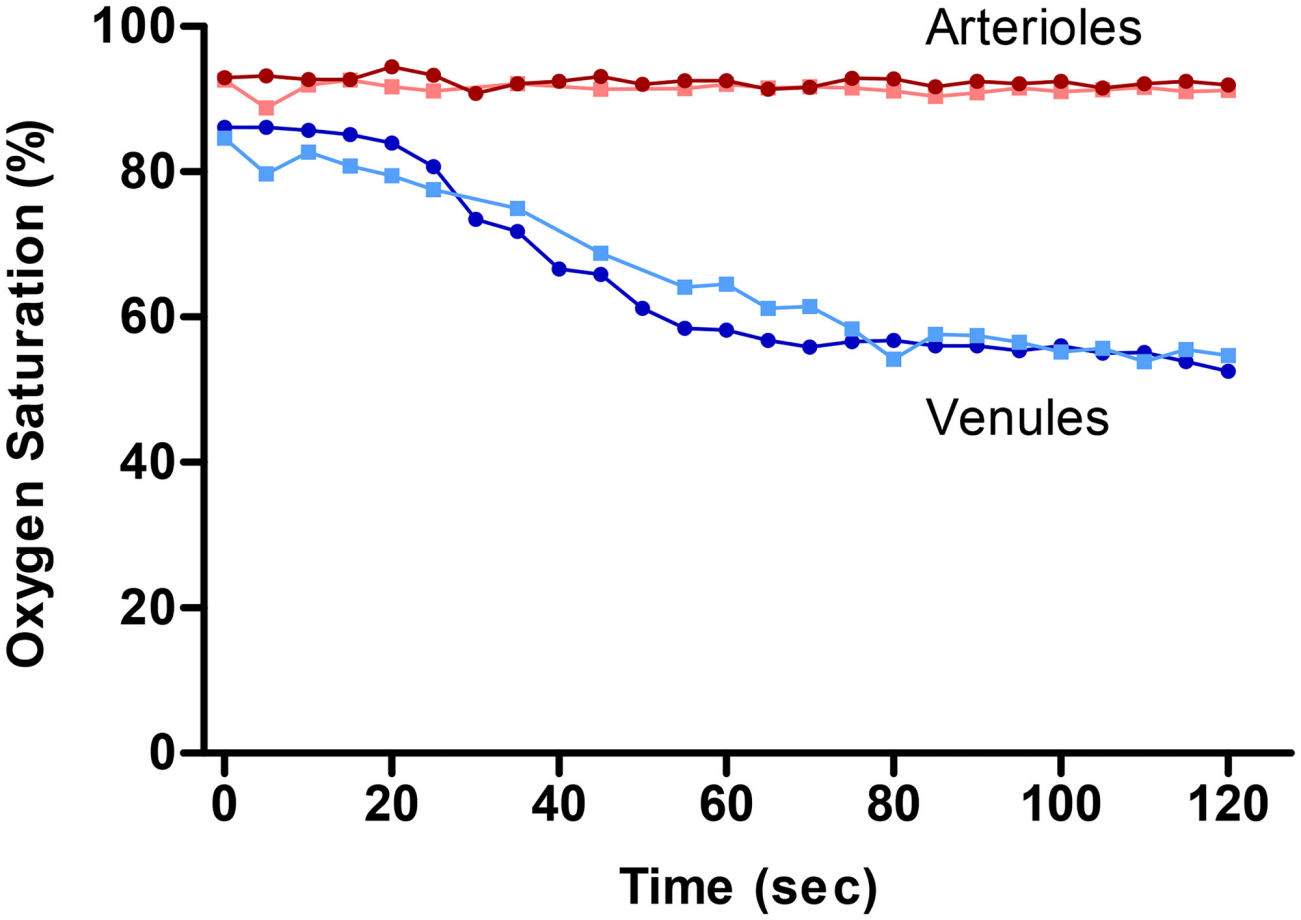
Retinal vessel oxygen saturation over time in arterioles (red) and venules (blue) in two healthy subjects. Subject 1 is denoted with circles in dark red and dark blue, subject 2 is denoted with squares in lighter red and lighter blue. Measurements were performed every five seconds during the first 120 seconds of recovery time after 100% oxygen breathing.

In two healthy individuals, retinal oxygen saturation in arterioles and venules was monitored every five seconds for two minutes after hyperoxia and the oxygen saturation recovers back to baseline values within two minutes (Fig 3).

## Discussion

Retinal vessel oxygen saturation increases modestly in arterioles and dramatically in venules during 100% oxygen breathing and the AV difference decreases. The vessels constrict during 100% oxygen breathing.

The modest increase in arterioles and dramatic increase in venules reflects the unusual anatomy and physiology of the retina. The saturation in the arterioles rises only slightly with hyperoxia as the saturation cannot go above 100%. The retinal AV difference in oxygen saturation is dramatically decreased during 100% oxygen breathing compared to normoxic levels (Table 2). This reflects a large oxygen influx from the choroid to the retina and reduced oxygen contribution by the retinal circulation to meet retinal tissue demand. The retinal arterioles constrict by 6.7% as vessel regulation reduces retinal blood flow to counter the enormous inflow of oxygen from the choroid over the retinal layers during pure oxygen breathing. According to the law of Hagen—Poiseuille, the resistance to blood flow is related to the fourth power of the diameter of a vessel. Oxygen extraction is the product of blood flow and AV difference in oxygen saturation so oxygen extraction by the retinal circulation is decreased during 100% oxygen breathing. That does, however, not mean that oxygenation of the inner layer of the retinal tissue is decreased during 100% oxygen breathing. The decrease has been made up by an increased oxygen contribution by the choroid. The regulation of blood flow during 100% oxygen breathing in the choroid is limited compared to the retina [5, 15]. Therefore, when the system receives much more oxygen during 100% oxygen breathing, the reaction in choroidal vessels is not the same as in retinal vessels, where the diameter in retinal vessels decreases during pure oxygen breathing. This leads to an increased oxygen contribution by the choroid to the inner retina. Even if the oxygen extraction from the retinal circulation is decreased, the total extraction of oxygen from the retinal and choroidal circulation is not necessarily decreased. The same pattern has been shown in various invasive animal studies [9–11, 16–18]. The increase in retinal venous oxygen saturation can therefore be explained by the oxygen flux from the choroid through the retina.

The increased oxygen saturation in venules corresponds to an increase in PO_2_ from 27.4 mmHg to 41.5 mmHg and it may be presumed that the inner retinal PO_2_ is at least that high. The hyperoxic PO_2_ values are similar to those measured over optic nerve head in pigs during 100% oxygen breathing (50.7±29.3mmHg)[19]. The normoxic PO_2_ values are also similar to what has been measured over the optic nerve head in pigs (24.1±11.6mmHg)[19] and the inner retina of cats (22.5±27.5mmHg).[20] No studies exist on PO_2_ measurements in healthy human eyes because of the invasive nature of the measurement techniques. In the vitreous adjacent to the retina, PO_2_ has been measured 15.0±5.7mmHg [21] and 9.78±0.64mmHg [22] in individuals with macular holes and epiretinal membranes. These values are lower than what we measured.

Changes in vessel diameter and blood flow during 100% oxygen breathing have previously been measured in healthy humans. In agreement with our study, Tayyari et al.[23] found that retinal arterioles diameter decreased during hyperoxia and blood flow decreased compared to their baseline and recovery measurements. Jean-Louis et al.[24] measured approximately 9% vasoconstriction in the arterioles and 14% constriction in venules in each of the four retinal quadrants during hyperoxia. Other studies show a similar outcome, Pakola and Grunwald measured 14.1% reduction in vein diameter and 56.4% decrease in blood flow during 100% oxygen breathing [7]. Riva et al. measured decreased retinal vein diameter by 12% during pure oxygen breathing. They also found that retinal blood flow was 60% less during pure oxygen breathing [8]. Palkovits et al.[25] also measured decrease in vessel diameter during pure oxygen breathing; the venules decreased by 13.0% in diameter in their study and the arteriolar diameter decreased by 12.1%, Kiss et al. measured 11–12% constriction in arterioles and 13–15% constriction in venules during hyperoxia [26]. Finally Rose and Hudson [27] measured 8.7% constriction in arterioles and 14% constriction in venules during carbogen (95% oxygen and 5% carbondioxide). Venules seem to constrict by similar values during hyperoxia in all the studies. However, the arterioles differ in constriction between the studies. In some studies [24, 27] they constrict less compared to the venules, which is the case in our study. In other studies the arterioles and venules constrict to the same degree [25, 26]. We do not know why the arterioles do not constrict to the same amount as the venules and we also do not know why they differ so much between the studies mentioned above. It could be due to technical issues. Also the pulsatile nature of arterioles could make it difficult to measure exact constriction. Rose and Hudson [27] conclude that when measuring diameter (or blood velocity) alone, one might conclude that the autoregulatory responses of the two types might differ but simultaneous velocity and diameter measurements as well as flow derivation show that the overall autoregulatory response is similar in magnitude in retinal arterioles and venules.

Hardarson et al.[28] performed 100% oxygen breathing experiment with an older version of the oximeter (prototype 2), obtaining similar oxygen saturation in venules as in this study but the arterioles measured higher in their experiment. Beach et al.[13] also measured the effect of pure oxygen breathing with a dual-wavelength oximeter where the venules increased on average by 19.2% in oxygen saturation during pure oxygen breathing, which is similar to our results. Palkovits et al.[25] recently measured retinal oxygen saturation in healthy individuals after pure oxygen breathing for 30 minutes but with a different type of oximeter (Imedos GmbH). Their arteriolar oxygen saturation values were similar to ours during baseline but slightly higher by almost two percentage points during pure oxygen breathing. They measured the venules higher in oxygen saturation during baseline by 10 percentage points compared to our measurements but similar during pure oxygen breathing.

As can be seen in Fig 3, oxygen saturation seems to recover from hyperoxic levels to normoxic levels within two minutes. The oxygen saturation numbers from the two healthy individuals compare well with each other. The oxygen saturation in both arterioles and venules during baseline and recovery compare well and therefore indicate that the oximeter measures stable baselines. From the data, it is also clear that the oximeter is sensitive to changes in oxygen saturation as well as vessel diameter. The oximeter also measures incremental decreases in saturation during recovery after systemic hyperoxia. Finally, the baseline oxygen saturation values compare well to previous studies done with the same type of oximeter following the same baseline measurement protocol [12, 29].

Our study has some limitations. The technique is based on reflected light and calculations on oxygen saturation and therefore the oxygen saturation values are relative but not absolute which must be kept in mind when reading through the data. Also, the fact that arterial oxygen saturation only reaches about 94.6% in oxygen saturation during 100% oxygen breathing could imply that there is some error in calibration of the oximeter. Finger pulse oximetry increased from 97% in baseline value to 99% during 100% oxygen breathing. The increase in retinal arteriolar oxygen saturation is similar but the exact pulse oximetry values are somewhat higher. It is unknown whether 100% oxygen breathing results in values of 100% oxygen saturation of the arterioles with the oximeter used in the study but comparison with pulse oximetry points to a calibration error of the oximeter. Palkovits et al.[25] found similar results when they measured the effect of hyperoxia with a different type of oximeter (from Imedos GmbH) where the arterioles did not reach 100% oxygen saturation during hyperoxia. They speculated that the cause could be either a calibration error of the oximeter, or there could be countercurrent exchange between the central retinal artery and vein. The calibration of the Oxymap T1 and Imedos oximeter are both based on reference data obtained from oxygen saturation measurements by Schweitzer et al. where the mean oxygen saturation measured in healthy individuals during normal room air breathing was 92.2% in retinal arterioles and 57.9% in venules.

In summary, retinal oxygen saturation increased modestly in arterioles and dramatically in venules and the AV difference decreased during 100% oxygen breathing. This reflects the unusual anatomy and physiology of the eye with a huge oxygen flux from the choroid.

## Supporting Information

S1 File Specific data for all individuals and data for kinetics. (XLSX)
